# Gender modifies the effect of body mass index on lung function decline in mild-to-moderate COPD patients: a pooled analysis

**DOI:** 10.1186/s12931-021-01656-5

**Published:** 2021-02-18

**Authors:** Wenjia Chen, Mohsen Sadatsafavi, J. Mark FitzGerald, Larry D. Lynd, Don D. Sin

**Affiliations:** 1 Respiratory Evaluation Sciences Program, Collaboration for Outcomes Research and Evaluation, Faculty of Pharmaceutical Sciences, University of British Columbia, 2405 Wesbrook Mall, Vancouver, BC V6T 1Z3 Canada; 2 UBC Centre for Heart Lung Innovation, St Paul’s Hospital, Providence Building, Room 8446, 1081 Burrard Street, Vancouver, BC V6Z 1Y6 Canada; 3 Division of Respiratory Medicine, Faculty of Medicine, The University of British Columbia, Vancouver, Canada; 4 Centre for Lung Health, Vancouver Coastal Health Research Institute, University of British Columbia, 7th Floor, 2775 Laurel Street, Vancouver, BC V5Z 1M9 Canada; 5 Centre for Health Evaluation and Outcome Sciences, The University of British Columbia, Vancouver, Canada

**Keywords:** Body mass index, FEV1, Lung function decline, COPD

## Abstract

**Background:**

Low body weight is associated with poor prognosis in patients with chronic obstructive pulmonary disease (COPD). However, it is not known whether gender modifies this relationship.

**Methods:**

We pooled data of 8686 COPD patients from 7 studies with a median length of 36-months of follow up. Using a longitudinal natural cubic spline regression model, we examined the dose–response relationship between body mass index (BMI) and the rate of decline in forced expiratory volume in one second (FEV_1_) in patients with GOLD 1 and 2 disease, stratified by gender and adjusted for age, smoking status, and cohort effects.

**Results:**

There was an inverse linear relationship between BMI and the rate of FEV_1_ decline in GOLD Grades 1 and 2, which was modified by gender (p < 0.001). In male patients, an increase of BMI by 1 kg/m^2^ reduced FEV_1_ decline by 1.05 mL/year (95% CI 0.96, 1.14). However, in female patients, BMI status did not have a clinically meaningful impact on FEV_1_ decline: an increase of baseline BMI by 1 kg/m^2^ reduced FEV_1_ decline by 0.16 ml/year (95% CI 0.11, 0.21). These gender-modified relationships were similar between GOLD 1 and 2 patients, and between current and former smokers.

**Conclusion:**

In mild to moderate COPD, higher BMI was associated with a less rapid decline of FEV_1_ in male patients whereas this association was minimal in females patients. This gender-specific BMI effect was independent of COPD severity and smoking status.

## Background

Chronic obstructive pulmonary disease (COPD) is one of the leading causes of morbidity and mortality in the world [1]. According to the World Health Organization, over the next 20 years COPD will rise from the fifth to third leading cause of death worldwide [2]. Among newly diagnosed COPD patients, over half had mild disease, one third had moderate, and 10% of patients had severe or very severe disease [3]. The progression of COPD is characterized by an accelerated decline in lung function as indicated by forced expiratory volume in one second (FEV_1_). On average, patients with mild to moderate COPD experience a faster decline in FEV1 over time than those with the more severe form of COPD [4]. Modification of lung function trajectory early in its course provides an opportunity to ameliorate patient’s quality of life and extend their life expectancy. It is now well-recognized that cachexia is a significant risk factor for poor outcomes including mortality in COPD patients [6–8]. However, while multiple studies report that those with low body mass index (BMI) may be at risk of COPD progression and those who are obese may be protected [5, 6], others have shown no significant association between BMI and FEV_1_ decline [11, 12]. One reason for the controversy is the fact that a decline in lung function varies according to certain factors, most notably smoking, gender, and disease stage [5–7]. Knowing whether BMI is a significant risk factor for COPD progression in patients with mild and moderate COPD is important, because it represents a potentially modifiable trait. Thus, we pooled individual-level data from 7 large international studies into a single, combined dataset, and examined the dose–response relationship between BMI and the rate of FEV_1_ decline in patients with mild and moderate COPD, according to gender and smoking status.

## Methods

### Study design and settings

This was a pooled analysis of patient-level data from 6 randomized controlled trials (RCTs) and 1 non-interventional prospective study. The RCTs were part of the Inhaled Steroids Effect Evaluation in COPD study (ISEEC) [7] and included the Lung Health Study (LHS, n = 5 594 patients, 11 years of follow-up) [8], the European Respiratory Society study on COPD (EUROSCOP, n = 1039 patients, 3 years of follow-up) [9], Inhaled Steroids in Obstructive Lung Disease in Europe (ISOLDE, n = 591 patients, 3 years of follow-up) [10], Copenhagen City Lung Study (CCLS, n = 225 patients, 3 years) [11], studies by Calverley et al. (n = 336 patients, 12 months of follow-up) [12] and Szafranski et al. (n = 292 patients, 12 months of follow-up) [13]. We additionally included data from a non-RCT, the Evaluation of COPD Longitudinally to Identify Predictive Surrogate Endpoints (ECLIPSE) study [14], which was a 3-year non-interventional study of 2652 patients with stable COPD from 46 centers across 12 countries [15]. By pooling data from these 7 cohorts, the final study sample included patients with mostly mild and moderate COPD according to the Global Initiative for Chronic Obstructive Lung Disease (GOLD) severity grades at baseline (GOLD Grade 1, FEV_1_ ≥ 80 percent of predicted at 25 years of age, mild COPD; and Grade 2, FEV_1_ 50–79 percent predicted, moderate COPD). We excluded patients in GOLD Grades 3 and 4 because they demonstrated different FEV_1_ trajectories over time compared with GOLD 1 and 2 patients (Table S1). For parsimony, we combined GOLD Grades 1 and 2 into one group because their FEV_1_ decline rates were similar (see Additional file 1: Table S1 and Figures S1, S2 for the observed rate of FEV_1_ decline between GOLD Grades 1 and 2). Further, patients were included if they had a valid measurement of BMI (≥ 10 kg/m^2^) at baseline, and 3 or more measurements of FEV_1_ across 3 or more different time points, which enabled a stable estimate of the FEV_1_ decline slope. Of note, spirometry standards and quality assurance were similar between the ISEEC trials and ECLIPSE. The Hankinson’s prediction equation was used to calculate percent of predicted FEV_1_ across all studies, using reference values for Caucasians, African–Americans, and Mexican–Americans derived from 7429 asymptomatic, non-smoking participants in the Third National Health and Nutrition Examination Survey (NHANES III) [16]. Because the provision of inhaled corticosteroids (ICS) did not affect FEV_1_ decline over time in ISEEC [7], this treatment intervention was not considered a confounder in the present study. Thus, we included patients from both the treatment and placebo arms of the RCTs.

### Study variables

The primary outcome was the change in the absolute value of post-bronchodilator FEV_1_ over time, which was assessed in all included studies through standardized spirometry measurements as described previously [7]. We did not evaluate changes in the percent predicted FEV_1_ over time because this parameter is adjusted for height, which is a key component of the BMI measurement.

The primary exposure, BMI at the baseline visit, was expressed as a continuous variable, and was obtained by dividing patient’s weight (Kg) by height squared (m^2^). We also examined the effects of gender (male, female) and their 2-way interaction effects with BMI, controlling for the confounding effect of cigarette smoking. As there could be significant between-study heterogeneity in the distribution of risk factors, study design, laboratory protocols, and enrollment period across the individual studies, we included relevant patient-level covariates in the model. This included baseline age as a continuous variable, follow-up years, and a categorical variable indicating cohort membership to account for other unobserved between-study heterogeneity.

### Statistical analysis

All analyses were performed using SAS 9.3 (SAS Institute Inc, Cary, NC, United States). Detailed descriptions of the statistical methods are provided in Additional file 1: Sect. 1. The criterion for statistical significance was a two-tailed p-value of 0.05 or less. Descriptive statistics were calculated, and comparisons were made using Pearson Chi-square tests for categorical variables and Kruskal–Wallis tests for continuous variables.

First, to examine the unadjusted relationship between BMI and the rate of FEV_1_ decline, we fitted a linear mixed-effects model with two predictors: a random intercept (corresponding to baseline FEV_1_ value) and a random slope of follow-up time (corresponding to the rate of FEV_1_ decline). From this analysis, we obtained the individual rate of FEV_1_ decline (i.e., the random slope), and plotted it against the individual’s baseline BMI value in a scatter plot.

Next, to evaluate the covariate-adjusted, averaged, potentially non-linear relationship between BMI and the rate of FEV_1_ decline, we applied natural cubic spline models. This longitudinal regression model had FEV_1_ values as the dependent variable, and contained the following independent variables: a spline function of BMI (primary exposure in which knots were placed across every 5th percentile), age, gender, smoking status, follow-up years, cohort status, and 2nd-order and 3rd-oder interactions terms between BMI, gender, smoking status, and follow-up years. We did not include an interaction term between BMI and age because based on a preliminary variable selection process, we found that its inclusion reduced model fit. These natural cubic splines produced smooth curves, which took into account the nonlinear components of the relationship between the exposure variables and the outcome. Next, outcomes were derived as the covariate-adjusted dose–response curves of BMI-rate of FEV_1_ decline using a robust causal inference technique named the G-computation [17]. In specific, based on the regression results, we obtained the individual FEV_1_ values at the 12-month follow-up, and calculated the individual rate of FEV_1_ decline as the instantaneous rate of change in FEV_1_ over a minimal duration of time (0.5E−5). We then evaluated the averaged changes in the rate of FEV_1_ decline per unit increase in baseline BMI value in dose–response curves according to gender. To enable the construction of 95% confidence bands of the dose–response curves, which accounted for the longitudinal, correlated data structure, we applied 1000 rounds of bootstrapping, which has been previously shown to efficiently handle correlated time-series data [18]. In a secondary analysis, the results were further stratified by smoking status to determine the impact of smoking on the relationship between BMI and FEV_1_ decline.

## Results

### Characteristics of the study population

**Fig. 1:**
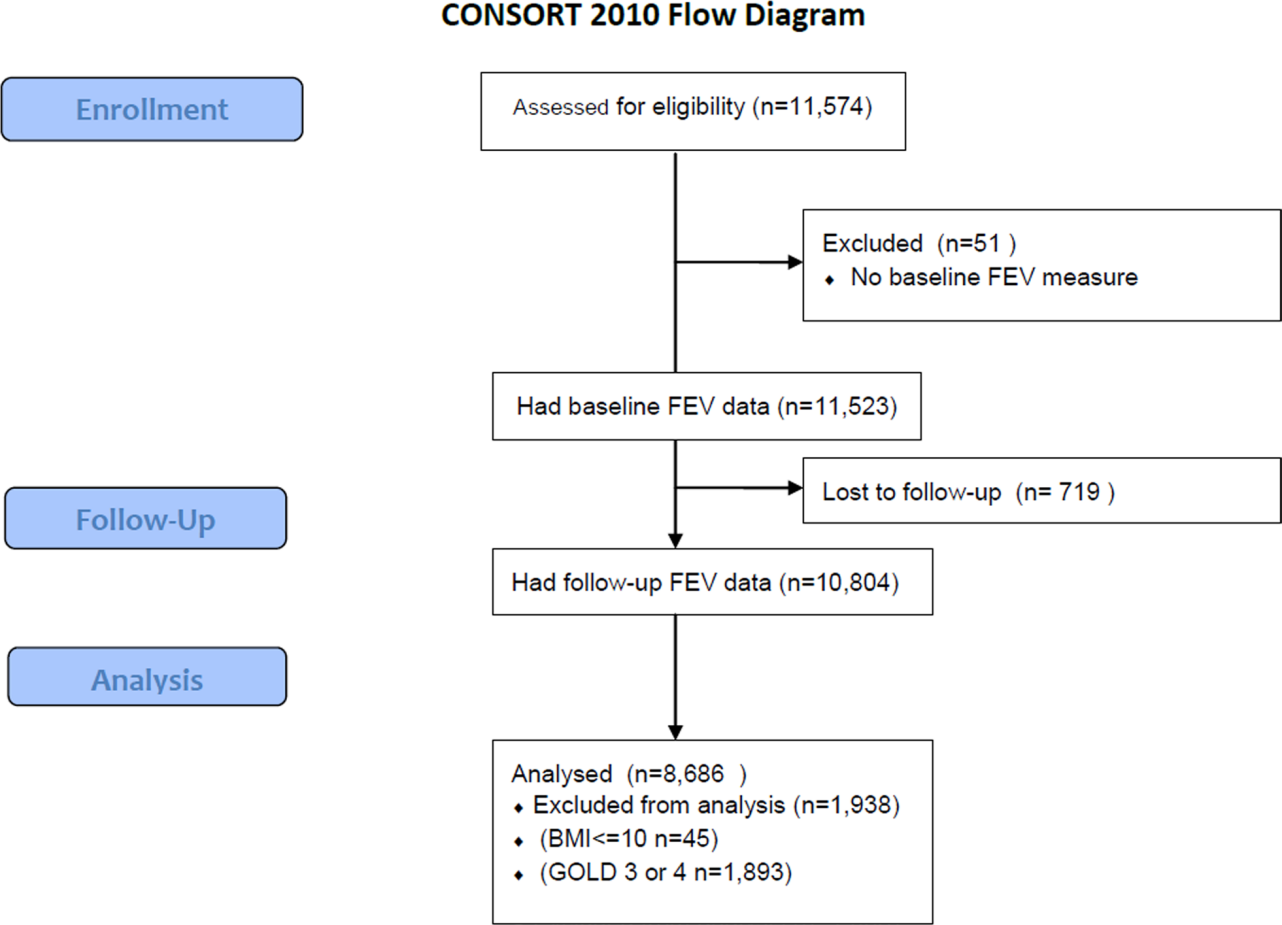
Flowchart of cohort selection

This pooled analysis comprised of 8686 COPD patients (Fig. 1, cohort selection). Table 1 presents the baseline characteristics of the study population. The mean baseline age was 51.9 years (SD = 9.1); 37% were women; and 56% were current smokers. The average BMI at baseline was 25.7 kg/m^2^ (SD = 4.3). The median follow-up time was 36 months. A total of 3674 (42%) patients were in GOLD Grade 1; 5012 (58%) were in GOLD Grade 2.

**Table 1:** Baseline characteristics of the study population

	Total	Calverley	CCLS	ECLIPSE	EUROSCOPE	ISOLDE	LHS	Szafranski
Patient, N (%)	8686 (100)	90 (1.0)	231 (2.7)	1500 (17.3)	982 (11.3)	260 (3.0)	5591 (64.4)	32 (0.4)
Age, y (SD)	51.9 (9.1)	62.4 (9.4)	58.9 (9.1)	60.2 (9.0)	52.5 (7.6)	63.9 (8.0)	48.4 (6.8)	66.3 (8.5)
Women, N (%)	3229 (37.2)	25 (27.8)	94 (40.7)	665 (44.3)	276 (28.1)	81 (31.2)	2085 (37.3)	3 (9)
Current smoker, N (%)	4849 (55.9)	29 (32.2)	175 (75.8)	426 (28.7)	982 (100)	126 (48.5)	3096 (55.4)	15 (46.9)
GOLD grade, N (%)								
1	3674 (42.3)	12 (13.3)	105 (45.5)	575 (38.3)	354 (36.0)	9 (3.5)	2619 (46.8)	0 (0)
2	5012 (57.7)	78 (86.7)	126 (54.5)	925 (61.7)	628 (64.0)	251 (96.5)	2972 (53.2)	32 (100)
FEV_1_								
Absolute, Litre	2.62 (0.73)	1.84 (0.48)	2.54 (0.80)	2.35 (0.96)	2.60 (0.62)	1.83 (0.44)	2.75 (0.63)	1.62 (0.29)
% predicted	77.8 (14.4)	63.7 (13.5)	79.6 (15.6)	81.6 (25.6)	74.6 (11.6)	61.9 (8.6)	78.4 (9.1)	56.0 (6.0)
BMI, kg/m^2^	25.7 (4.3)	25.9 (5.3)	25.5 (4.2)	27.3 (5.4)	24.5 (3.3)	25.2 (4.3)	25.5 (3.9)	26.0 (5.3)
BMI category, N (%)								
Underweight (BMI < 18.5)	163 (1.9)	5 (5.6)	3 (1.3)	30 (2.0)	21 (2.1)	13 (5.0)	90 (1.6)	1 (3.1)
Normal (BMI 18.5–25.0)	3945 (45.4)	41 (45.6)	118 (50.1)	510 (34.0)	555 (56.5)	118 (45.4)	2589 (46.3)	14 (43.8)
Overweight (BMI 25.1–30.0)	3340 (38.5)	27 (30.0)	71 (30.7)	589 (39.3)	354 (30.0)	93 (35.8)	2194 (39.2)	12 (37.5)
Obese (BMI > 30.1)	1238 (14.3)	17 (18.9)	39 (16.9)	371 (24.7)	52 (5.3)	36 (13.8)	718 (12.8)	5 (15.6)
Follow-up, mo	36 (median)							

*BMI* body mass index, *CCLS* City Lung Study, *ECLIPSE* Evaluation of COPD Longitudinally to Identify Predictive Surrogate Endpoints Study, *EUROSCOPE* European Respiratory Society study on COPD, *GOLD* Global Initiative for Chronic Obstructive Lung Disease (GOLD) spirometric grades, *ISOLDE* Inhaled Steroids in Obstructive Lung Disease in Europe, *LHS* lung health study, *mo* moth, *N* number, *SD* standard deviation, *y*, year

Table 2 presents the gender-specific baseline characteristics and the observed rate of FEV_1_ decline of the combined GOLD Grades 1 and 2 samples. Male patients experienced a significantly faster decline in absolute FEV_1_ values compared to female patients (mL/year, − 36.6 vs − 29.2, p-value < 0.001). Male and female patients had similar mean ages (51.8 vs. 51.9 years) and proportionality of current smokers (54% vs. 57%). However, the majority of males had normal body weight (58% of BMI between 18.5 and 25.0 kg/m^2^), whereas the majority of females were more likely to be overweight or obese (60% of had a BMI above 25.1 kg/m^2^). Additional file 1: Table S2 shows the observed baseline FEV_1_ and the rate of FEV_1_ decline by smoking status and BMI level. Underweight individuals (BMI < 18.5 kg/m^2^) had a lower baseline FEV_1_ compared to other BMI groups (L, 2.17 vs 2.56–2.73), while obese individuals (BMI ≥ 30.1 kg/m^2^) had a slower rate of decline than those who were overweight (BMI 25.1–30.0 kg/m^2^), normal (BMI 18.5–25.0 kg/m^2^) or underweight (mL/year, − 27.5 vs − 34.4, − 35.3, − 34.6, respectively). Current smokers had a much more rapid decline than ex-smokers (mL/year, − 40.9 vs − 24.9).

**Table 2:** Observed rate of FEV_1_ decline according to GOLD subgroups, gender, smoking status and BMI category

	GOLD Grades 1 and 2		
	Male (N = 5457)	Female (N = 3229)	p-value^*^
Rate of FEV_1_ decline, mean (95% CI), mL/year	− 36.6 (− 37.6, − 35.6)	− 29.2 (− 30.2, − 28.1)	p < 0.001
Age, y, mean (SD)	51.8 (8.8)	51.9 (9.3)	p = 0.40
BMI, kg/m^2^, mean(SD)	26.3 (3.9)	24.8 (4.7)	p < 0.001
BMI category, n (%)			p < 0.001
Underweight (BMI < 18.5)	54 (1.0)	109 (3.4)	
Normal (BMI 18.5–25.0)	2082 (38.2)	1,863 (57.7)	
Overweight (BMI 25.1–30.0)	2477 (45.4)	863 (26.7)	
Obese (BMI > 30.1)	844 (15.5)	394 (12.2)	
Smoking status, n (%)			p = 0.02
Ex-smoker	1472 (45.7)	2350 (43.1)	
Current smoker	1750 (54.3)	3099 (56.9)	

*BMI* body mass index, *GOLD* Global Initiative for Chronic Obstructive Lung Disease (GOLD) spirometric grades, *N* number, *SD* standard deviation

^*^p-values were obtained from Kruskal–Wallis test for continuous variables and Pearson chi-square test for categorical variables

### Gender-modified effects of BMI on the rate of FEV_1_ decline

**Fig. 2:**
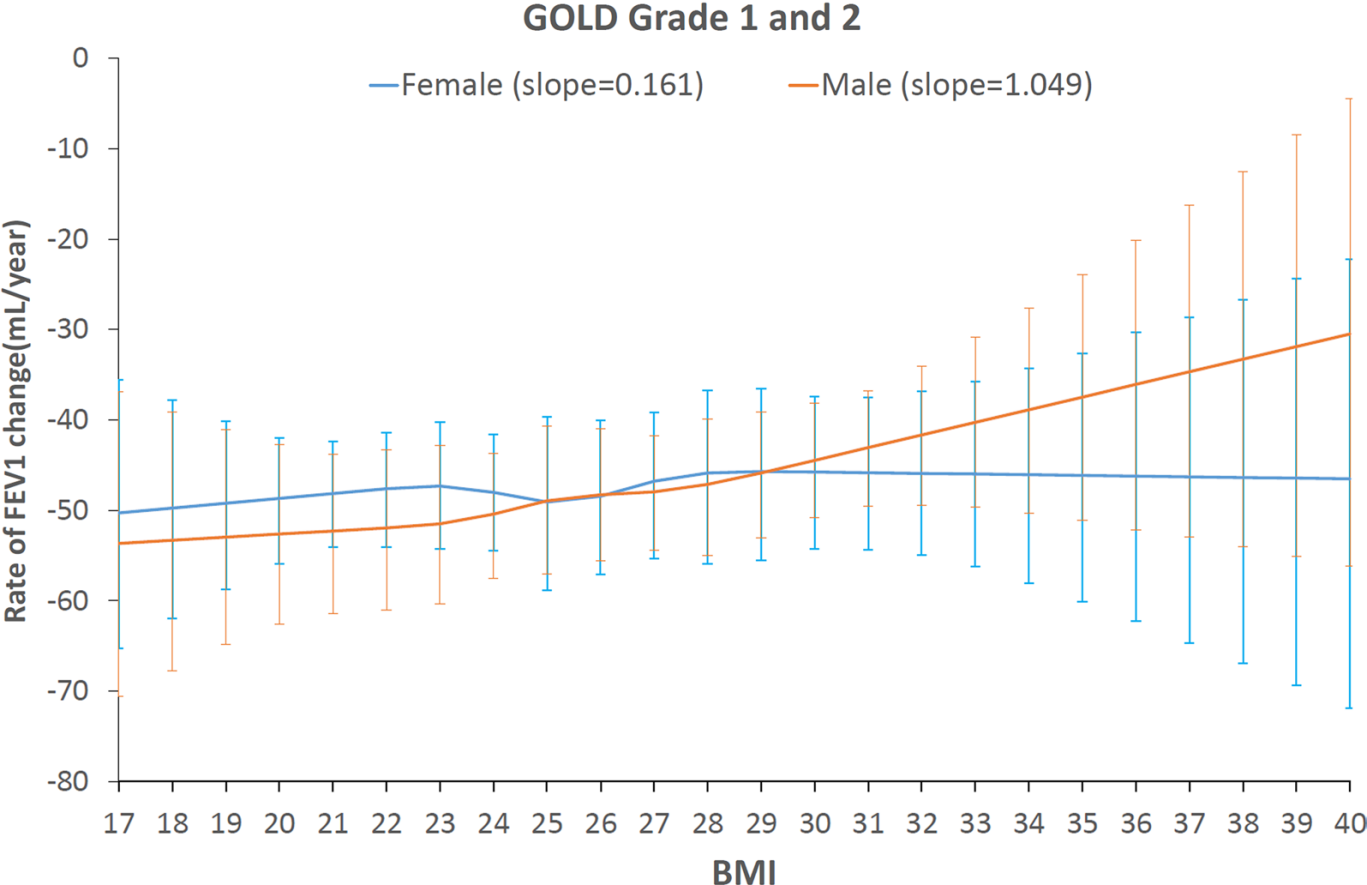
Diagrammatic representation of the dose–response curves of the effect of baseline BMI values on the rate of FEV_1_ decline in absolute values (mL/year) in GOLD Grades 1 and 2. The blue curve is averaged association of female patients, the orange curve is averaged association of male patients. The slope refers to reduction in rate of FEV_1_ decline per 1 kg/m^2^ increase in baseline BMI

**Figure ****2** illustrates the fitted dose–response relationship between BMI (x-axis) and the rate of decline in absolute FEV_1_ values (y-axis, with confidence intervals shown in error bars) in combined GOLD Grades 1 and 2. The confidence interval of the curve was wider at both ends of the BMI scale, reflecting a greater variance of the association at extreme BMI values, probably due to the smaller number of patients at the extremes.

Gender significantly modified the relationship between BMI and FEV_1_ decline (p < 0.001). In male patients, the dose–response curve depicted a reverse association between BMI and rate of decline in absolute FEV_1_ values. This relationship was mostly linear, except for a slight fluctuation in the line at a BMI of 25 kg/m^2^ (Fig. 2). The slope of this curve showed that an increase of BMI by 1 kg/m^2^ reduced FEV_1_ decline by approximately 1.05 mL/year (95% CI: 0.96, 1.14). In female patients, the slope of the curve showed that BMI had a very small (and clinically insignificant) effect: an increase of BMI of 1 kg/m^2^ reduced FEV_1_ decline by only 0.16 ml/year (95% CI: 0.11, 0.21).

**Fig. 3:**
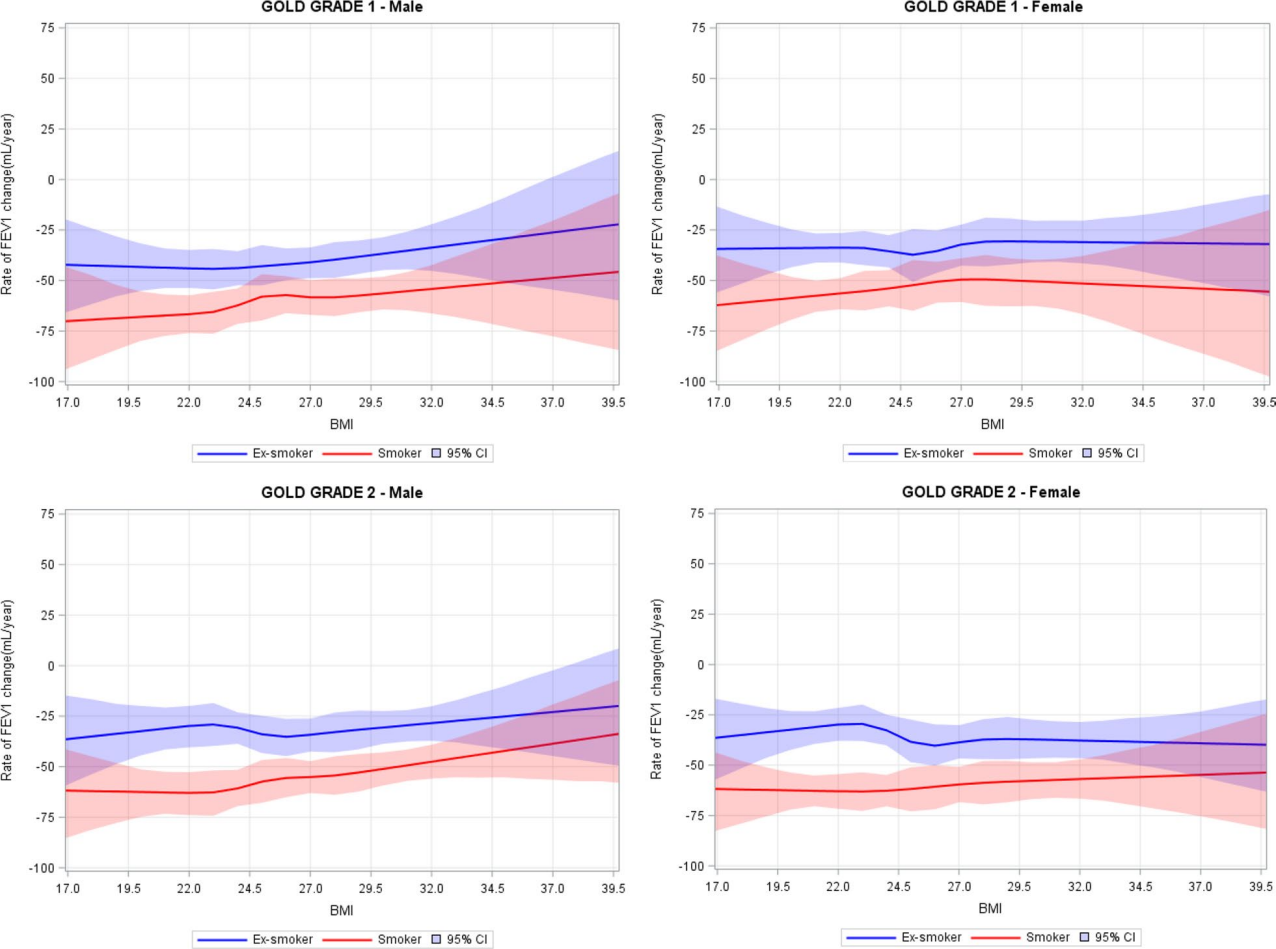
Diagrammatic representation of the dose–response curves of the effect of baseline BMI values on the rate of FEV_1_ decline in absolute values (mL/year), stratified across GOLD grades, gender, and smoking status. Upper left panel, male patients within GOLD Grade 1, upper right panel, female patients within GOLD Grade 1, lower left panel, male patients within GOLD Grade 2, lower right panel, female patients within GOLD Grade 2. The blue line is averaged estimates of ex-smokers, the red line is averaged estimates of smokers, the shaded area denotes 95% credible interval

In a secondary analysis, the effects of BMI were stratified by GOLD Grades and smoking status (Fig. 3, upper panel, male patients [left, GOLD 1; right, GOLD 2], lower panel, female patients [left, GOLD 1, right, GOLD 2]). As for the effects of smoking, within GOLD Grade 1, the gender-specific dose–response curves appeared in parallel between current smokers and ex-smokers, though the curves were statistically different from each other (p < 0.001). Within GOLD Grade 2, there was a notable narrowing of the gap at higher BMI levels between current smokers and ex-smokers. This suggests that, conditional on COPD severity and gender, the additional impact of smoking on the relationship between BMI and FEV_1_ decline was small in GOLD Grade 1, whereas higher BMI level appeared to have a slight protective effect on FEV_1_ decline in GOLD Grade 2 current smokers, in particular among male smokers. Of note, the highest risk of decline was observed in underweight male smokers with GOLD 1 disease, who experienced, on average > 70 ml/year decline in FEV_1_ (Fig. 3).

## Discussion

In a large pooled analysis of seven multinational prospective studies, we showed that the relationship between BMI and FEV_1_ decline in mild to moderate COPD was significantly modified by gender. In females, BMI had no material impact on FEV_1_ decline; whereas in males, a 1 kg/m^2^ increase in BMI was associated with a reduction of approximately 1 mL/year in the rate of decline in absolute FEV_1_ values. The protective effect of high BMI appeared to be more prominent in GOLD 2 male smokers. Of note, underweight male current smokers in GOLD 1 were at the highest risk of disease progression. We did not include percent of predicted FEV_1_ as an outcome given that it was derived from height.

Previous studies have largely focused on effects of smoking as a risk factor for COPD disease progression. Consistent with previous findings, we found that smokers experienced over 10 mL/year faster decline in FEV_1_ compared with sustained quitters [19]. We extend these previous findings in this large pooled analysis by showing that BMI significantly impacts FEV_1_ decline in males but has only a minimal effect in females with mild to moderate COPD_._ For example, the annual decline of FEV_1_ was at least 6 mL/year faster in underweight (BMI < 19 kg/m^2^) male patients than those who were overweight (BMI ≥ 25 kg/m^2^).

The mechanisms by which BMI modifies FEV_1_ decline have not been fully elucidated. High BMI may represent better nutrition status [20], increased body fat, muscle mass, and/or bone mineral density [21], while lower BMI may indicate poor nutrition and skeletal muscle loss that leads to accelerated lung function loss [22]. The lower baseline FEV_1_ of underweight patients may also indicate childhood conditions such as restricted early-life growth that leads to poor lung development [23]. In addition, emphysema might have also played a role, because emphysema is significantly associated with reduced BMI [24], and is also more common in male patients [25]. Furthermore, sex-modified effects of BMI on FEV_1_ decline may be related to anatomical differences in the large airways that have been observed between men and women, such as smaller central airways and lower thoracic volume in females [26, 27]. Due to concerns regarding reverse causality, we were unable to study the effects of dynamic changes in BMI (i.e., increased lung burden caused weight loss). Notwithstanding these important mechanistic issues, BMI is easy to measure, accurate and reproducible. As such, BMI may be used clinically to identify COPD patients (particularly males) at risk for rapid disease progression.

A major strength of this analysis was that it pooled individual-level data from seven high-quality long-term studies, which reduced heterogeneity and yielded more reliable results than the previously reported meta-analysis [28]. The inclusion of ECLIPSE, a real-world prospective cohort, added to the external validity and generalisability of results. Another strength was the use of a robust and powerful statistical approach, which adapted longitudinal analyses to principles of restricted cubic splines. This enabled us to extend measures of static, cross-sectional dose–response relationship to more pragmatic metrics representing the impact of BMI on the progression of COPD (ie., the rate of FEV_1_ decline). Importantly, our analyses were stratified by gender and adjusted for various confounders including GOLD grades of severity, cohort and calendar effects, smoking status and its interactions with BMI. This enabled us to tease out the differential effects of BMI between genders, which were not well known previously.

Our findings need to be interpreted within the context of certain limitations. First, BMI is an approximate measure for nutritional status, because it is unable to distinguish between fat and fat-free mass or its distribution. Future studies should consider other anthropometric measurements in female patients with mild to moderate COPD and investigate the potential roles of muscle and fat mass in the gender-specific progression of COPD. Second, we assessed BMI at baseline and weight may change dynamically over time. This was not necessarily a limitation, because this “intention-to-treat” approach protects against reverse causality and provides more valid inference to our research question. Third, this analysis could not adjust for unrecorded potential confounders such as comorbidities and exacerbations. However, while the comorbidities of low BMI COPD patients are different from those of patients with a high BMI [29], the direct impact of comorbidities on lung function decline is largely unknown. Fourth, non-smokers were not included in this study as data were unavailable, which may limit the generalizability of results. Finally, we limited the sample to patients with 3 or more FEV_1_ measurements to evaluate a possible dose–response curve using a cubic spline analysis. Only 3% of the entire cohort comprised of patients with 2 (or fewer) FEV_1_ measurements and these patients had shorter follow-up period compared to those with 3 or more FEV_1_ measurements (median follow up, 13 vs. 36 months), but the former has similar baseline FEV_1_ and rate of FEV_1_ decline compared to the latter whose follow-up period was up to 13 months.

## Conclusion

We conclude that reduced BMI is a significant risk factor for accelerated decline in lung function but is modified by gender. Underweight male smokers with GOLD 1 disease are at the highest risk of rapid COPD progression and thus should be followed closely and be strongly counseled for smoking cessation.

## Supplementary Information

**Additional file 1: Table S1.** Observed rate of FEV1 decline across GOLD grades of severity, gender and smoking status. **Table S2.** Observed rate of FEV1 decline across gender, smoking status and BMI category in combined GOLD Grades 1 and 2. **Figure S1.** Scatter plot of BMI on individual rate of FEV_1_ decline in GOLD Grades 1 and 2.

## Data Availability

The data that support the findings of this pooled study are available from the individual trials, including CCLS, ECLIPSE, EUROSCOP, ISOLDE, LHS, and studies conducted by Calverley et al., Szafranski et al., but restrictions apply to the availability of these data, which were used under license for the current study, and so are not publicly available. Data are however available from the authors upon reasonable request and with permission of these trials.
