# Supplementary material for: Gender modifies the effect of body mass index on lung function decline in mild-to-moderate COPD patients: a pooled analysis
Source: Respir Res. 2021 Feb 18;22:59. doi: 10.1186/s12931-021-01656-5 (PMC7891012; doi:10.1186/s12931-021-01656-5)
Supplement: Supplementary file 1 — Additional file 1: Table S1. Observed rate of FEV1 decline across GOLD grades of severity, gender and smoking status. Table S2. Observed rate of FEV1 decline across gender, smoking status and BMI category in combined GOLD Grades 1 and 2. Figure S1. Scatter plot of BMI on individual rate of FEV1 decline in GOLD Grades 1 and 2. [file 12931_2021_1656_MOESM1_ESM.docx]

Additional material

**The relationship between body mass index and lung function decline in COPD: a pooled analysis**

Authors: Wenjia Chen, Mohsen Sadatsafavi, J Mark FitzGerald, Don D. Sin

**Section 1. Description of a longitudinal dose-response analysis**

All analyses were performed using SAS 9.3 (SAS Institute Inc, Cary, NC, United States). In a preliminary unadjusted analysis, we found that the relationship between BMI and FEV_1_ decline was significantly modified by the severity of COPD. Patients under GOLD Grade 1 and 2 experienced a more rapid decline compared to patients under GOLD Grade 3 and 4. In the main analysis, we were focused on patients with mild and moderate COPD (GOLD Grade 1 and 2), and combined these patients into one group.

**Examine the nonlinear relationship between BMI and FEV_1_ in a longitudinal cubic spline model**

In patients with GOLD Grades 1 and 2, we applied a natural cubic spline model to study the dose-response relationship between BMI and the rate of FEV_1_ decline. This was a longitudinal linear regression model of FEV_1_ based on the following equation:

$${FEV}_{1t}=\beta_{0}+\beta_{1}Age+\beta_{2}Gender+\beta_{3}Smoking+\beta_{4}Spl\left( BMI \right)+\beta_{5}{Year}_{t}+\beta_{6}Cohort+\beta_{7}Sex\times Spl\left( BMI \right)+\beta_{8}Smoking\times Spl\left( BMI \right)+\beta_{9}Spl\left( BMI \right)\times Year+\beta_{10}Age\times{Year}_{t}+\beta_{11}Smoking\times{Year}_{t}+\beta_{12}Spl\left( BMI \right)\times Sex\times{Year}_{t}+\beta_{13}Spl\left( BMI \right)\times Smoking\times{Year}_{t}+\epsilon$$

Where ${FEV}_{1t}$ is the FEV_1_ value at year *t*, $Spl\left( BMI \right)$ is a spline function of BMI with knots placed in every 5 percentiles. Other interaction terms were purposefully removed to pertain the best model fit. To fit this longitudinal model in which data were clustered within each patient, we applied 1 000 rounds of bootstrapping with replacement on study participants. The bootstrap method has been shown to perform equally well in balanced or nearly balanced longitudinal data when comparing to methods that explicitly consider the correlation structure when fitting the model (e.g., generalized estimating equation, mixed-effects models)(1).

**Estimate the covariate-adjusted dose-response curve of BMI on the rate of FEV_1_ decline**

Based on the regression results, the rate of FEV_1_ decline was estimated as the instantaneous rate of change in FEV_1_ over minimal duration of time *h,* i.e., the derivative of the predicted FEV_1_, which was written as

$${Rate of FEV}_{1}Decline= \lim_{n\to\infty} (\frac{{FEV}_{1 t+h}-{FEV}_{1 t}}{h\to0})$$

Next, we estimated the averaged, covariate-adjusted dose-response relationship between BMI and the rate of FEV_1_ decline associated with male and female patients for the GOLD Grade 1 and 2 group. The estimation was done in a counterfactual framework where, within each subgroup, we first estimated the rates of FEV_1_ decline for each participant with a baseline BMI ranging from 17 kg/m^2^ to 40 kg/m^2^ (covering 99 percentile of the BMI distribution in our sample). Here we respectively assumed that the participant was a female and a male. In this way, we estimated the dose-response curve of BMI on the rate of FEV_1_ decline for female and male from the same individual. Meanwhile, other covariates were set at the observed value. As the next step, we averaged the estimated dose-response curves by gender across the two subgroup of GOLD grades. Using this specific causal inference technique namely the G-computation(2), assuming we have controlled all confounding covariates, we can interpret such covariate-adjusted dose-response curves as gender- and severity-modified. Inference was obtained from the abovementioned bootstrapping process.

In a sensitivity analysis, we repeated this causal inference process to further stratify the dose-response relationship by smoking status.

**References**

1. Feng Z, McLerran D, Grizzle J. A comparison of statistical methods for clustered data analysis with Gaussian error. *Stat Med* 1996;15:1793–1806.

2. Austin PC, Urbach DR. Using G-computation to estimate the effect of regionalization of surgical services on the absolute reduction in the occurrence of adverse patient outcomes. *Med Care* 2013;51:797–805.

**Table S1. Observed rate of FEV1 decline across GOLD grades of severity, gender and smoking status.**

|  | Rate of FEV_1_ decline, *mL/year*  Mean (95% confidence interval) | | | |
| --- | --- | --- | --- | --- |
|  | GOLD 1  (n=3674) | GOLD 2  (n=5 012) | GOLD 3  (n=1 588) | GOLD 4  (n=485) |
| Overall | -36·9 (-38·0, -36·0) | -31·4 (-32·4, -30·4) | -0·7 (-1·3, -0·2) | 0·3 (-1·1, 1·7) |
| Gender |  |  |  |  |
| Female | -33·6 (-35·2, -32·1) | -26·4 (-27·7, -25·0) | -7·7 (-8·4, -7·0) | -11·5 (-13·9, -9·2) |
| Male | -38·8 (-37·6, -35·6) | -34·4 (-35·7, -33·0) | 1·9 (1·3, 2·5) | 3·2 (1·7, 4·8) |
| Smoking status |  |  |  |  |
| Ex-smoker | -28·3 (-29·7, -26·8) | -21·8 (-23·1, -20·4) | -1·9 (-2·6, -1·3) | -1·0 ( -2·6, 0·6) |
| Current smoker | -44·0 (-45·5, -42·5) | -38·8 (-40·2, -37·4) | 1·4 (0·5, 2·3) | 5·2 (2·2, 8·3) |

**Table S2. Observed rate of FEV1 decline across gender, smoking status and BMI category in combined GOLD Grades 1 and 2.**

|  | Baseline FEV_1_  Mean (95% confidence interval) | | Rate of FEV_1_ decline Mean (95% confidence interval)  (n=8 686) | |
| --- | --- | --- | --- | --- |
|  | *mL* | *%predicted* | *mL/year* | *%predicted/year* |
| Overall | 2.62 (2.60, 2.63) | 77.8 (77.5, 78.1) | -33.8  (-34.6, -33.1) | -0.60  (-0.62, -0.58) |
| Gender |  |  |  |  |
| Female | 2.12 (2.10, 2.13) | 79.0 (78.4, 79.5) | -29.2  (-30.2, -28.1) | -0.72  (-0.76, -0.50) |
| Male | 2.92 (2.90, 2.93) | 77.1 (76.8, 77.5) | -36.6  (-37.6, -35.6) | -0.52  (-0.54, -0.50) |
| BMI category |  |  |  |  |
| Underweight  (BMI<18.5) | 2.17 (2.07, 2.26) | 72.4 (70.5, 74.3) | -34.6  (-40.0, -29.1) | -0.89  (-1.05, -0.73) |
| Normal  (BMI 18.5-25.0) | 2.56 (2.54, 2.58) | 77.9 (77.5, 78.3) | -35.3  (-36.4, -34.2) | -0.66  (-0.69, -0.63) |
| Overweight  (BMI 25.1-30.0) | 2.73 (2.71, 2.76) | 78.4 (77.9, 78.8) | -34.4  (-35.6, -33.2) | -0.54  (-0.57, -0.52) |
| Obese  (BMI>30.1) | 2.56 (2.52, 2.60) | 76.8 (75.9, 77.7) | -27.5  (-29.6, -25.4) | -0.49  (-0.54, -0.43) |
| Smoking status |  |  |  |  |
| Ex-smoker | 2.59 (2.56, 2.61) | 78.9 (78.4, 79.4) | -24.9  (-25.8, -23.9) | -0.37  (-0.40, -0.35) |
| Current smoker | 2.64 (2.63, 2.66) | 77.0 (76.6, 77.3) | -40.9  (-42.0, -39.9) | -0.77  (-0.80, -0.74) |

**Figure S1. Scatter plot of BMI on individual rate of FEV_1_ decline in GOLD Grades 1 and 2.**
